# Cytokine Polymorphisms, Their Influence and Levels in Brazilian Patients with Pulmonary Tuberculosis during Antituberculosis Treatment

**DOI:** 10.1155/2013/285094

**Published:** 2013-03-27

**Authors:** Eliana Peresi, Larissa Ragozo Cardoso Oliveira, Weber Laurentino da Silva, Érika Alessandra Pellison Nunes da Costa, João Pessoa Araujo, Jairo Aparecido Ayres, Maria Rita Parise Fortes, Edward A. Graviss, Ana Carla Pereira, Sueli Aparecida Calvi

**Affiliations:** ^1^Tropical Disease Department, Botucatu School of Medicine, UNESP, São Paulo State University, Botucatu, SP, Brazil; ^2^Departamento de Doenças Tropicais e Diagnóstico por Imagem, Faculdade de Medicina de Botucatu, UNESP, Rubião Júnior S/N, Botucatu 18618-970, SP, Brazil; ^3^Lauro de Souza Lima Institute, Bauru, SP, Brazil; ^4^Microbiology and Immunology Department, Bioscience Institute, UNESP, São Paulo State University, Botucatu, SP, Brazil; ^5^Nursing Department, Botucatu School of Medicine, UNESP, São Paulo State University, Botucatu, SP, Brazil; ^6^Dermatology and Radiotherapy Department, Botucatu School of Medicine, UNESP, São Paulo State University, Botucatu, SP, Brazil; ^7^The Methodist Hospital Research Institute, Houston, TX, USA

## Abstract

Cytokines play an essential role during active tuberculosis disease and cytokine genes have been described in association with altered cytokine levels. Therefore, the aim of this study was to verify if *IFNG, IL12B, TNF, IL17A, IL10, and TGFB1* gene polymorphisms influence the immune response of Brazilian patients with pulmonary tuberculosis (PTB) at different time points of antituberculosis treatment (T1, T2, and T3). Our results showed the following associations: *IFNG* +874 T allele and *IFNG* +2109 A allele with higher IFN-**γ** levels; *IL12B* +1188 C allele with higher IL-12 levels; *TNF* −308 A allele with higher TNF-**α** plasma levels in controls and mRNA levels in PTB patients at T1; *IL17A* A allele at rs7747909 with higher IL-17 levels; *IL10* −819 T allele with higher IL-10 levels; and *TGFB1* +29 CC genotype higher TGF-**β** plasma levels in PTB patients at T2. The present study suggests that *IFNG* +874T/A, *IFNG* +2109A/G, *IL12B* +1188A/C, *IL10* −819C/T, and *TGFB1* +21C/T are associated with differential cytokine levels in pulmonary tuberculosis patients and may play a role in the initiation and maintenance of acquired cellular immunity to tuberculosis and in the outcome of the active disease while on antituberculosis treatment.

## 1. Introduction

*Mycobacterium tuberculosis* (*M. tuberculosis*) is an intracellular obligate aerobic pathogen which has a predilection for the lungs [1]. Macrophages initiate phagocytosis of *M. tuberculosis* bacilli and regulate immune responses mediated by proinflammatory cytokines such as TNF-*α*. Effector T lymphocytes (T cells) and natural killer (NK) cells secrete IFN-*γ* which activate alveolar macrophages to produce reactive intermediates from nitrogen and oxygen, inhibiting growth and promoting mycobacteria death [2]. IL-12, produced mainly by macrophages and dendritic cells, has a key role in the immune response to *M. tuberculosis*, bridging innate and adaptive immunity. Moreover, IL-12 induces T cells and NK cells to produce proinflammatory cytokines such as IFN-*γ* and TNF-*α* while also regulating the production of IL-17 [2, 3]. A synergistic response of IFN-*γ*, IL-12, TNF-*α* and IL-17 activates macrophage, stimulating these cells to eliminate the intracellular pathogen, acting as a major effector mechanism of the cellular immune response [4].

Despite the protective effect of Th1 responses against *M. tuberculosis*, certain cytokines, such as TNF-*α*, are correlated with the immunopathogenesis of the disease [5]. To prevent tissue damage, active tuberculosis is associated with decreased Th1 and increased production and action of suppressing cytokines produced by Th2 and T regulatory (Treg) cells, IL-10, and TGF-*β*, respectively, which act by deactivating macrophages, modulating proinflammatory cytokines, and reducing the antigen presenting function of T cells [6]. TGF-*β* also participates in the induction of fibrosis, a hallmark presentation of tuberculosis disease [7].

The dynamics of tuberculosis (TB) disease is complex, as various aspects of the parasite-host interaction contribute to the occurrence and presentation of the outcome. In this scenario there is an important contribution of human genetic susceptibility to disease after exposure to *M. tuberculosis* [8–10]. Cytokines have a key role in the defense against mycobacteria and their genes might be considered candidates for host susceptibility to the onset of active TB disease.

The association between cytokines polymorphisms and human TB susceptibility has been reported in studies with *ex vivo* cytokine production in response to mycobacterial antigens and their correlation with variant genotypes; however few studies have investigated their role in modulating the overall cytokine response during PTB treatment [11–19]. We hypothesized that studying the actual overall immune cytokine pattern of PTB patients could be important to our understanding of active disease and possible establishment of biomarkers of recovery and anti-TB treatment efficiency. Therefore the aim of this study was to verify the influence of *IFNG*, *IL12B*, *TNF*, *IL17A*, *IL10*, and *TGFB1* gene polymorphisms on the overall cytokine response of Brazilian patients with PTB under anti-TB treatment.

## 2. Material and Methods

### 2.1. Study Population

The study group enrolled 31 Brazilian patients attending the Infectious and Parasitic Diseases Services at Botucatu Medical School University Hospital, UNESP, Botucatu Teaching Health Centre and Primary Healthcare Units of Botucatu and surrounding region with PTB diagnosis confirmed by sputum smear or culture positivity for *M. tuberculosis* or by clinical-epidemiologic data with laboratory and image exams (radiography or computerized tomography (CT)) compatible with active TB. Patients with PTB concurrent with other active granulomatous diseases or HIV were excluded. All patients diagnosed with PTB received treatment for six months, using the four first-line drugs: isoniazid, ethambutol, pyrazinamide, and rifampicin. For the evaluation of immunologic function, patients' samples were collected based on the anti-TB treatment timeline, defined as T1: after diagnosis and with no more than one month of treatment; T2: with three months of treatment; and T3: with six months of treatment. Patient-specimen distribution for each time point included T1 (*n* = 5); T2 (*n* = 1); T3 (*n* = 2); with two time points T1 and T2 (*n* = 4); and with the three time points T1, T2, and T3 (*n* = 19). For normal controls (C), we studied 20 health care workers from Botucatu Medical School (Botucatu, SP, Brazil), 9 males (mean age 40.4 years), and 11 females (mean age 34.1 years), without clinical complaints and with no history of TB disease, autoimmune disease, and other infectious disease. All controls were BCG vaccinated in childhood and tuberculin skin test (TST) positive (induration ≥ 5 mm). All patients and controls agreed to participate in the study, after study clarification and written informed consent.

### 2.2. Single Nucleotide Polymorphism (SNP) Genotyping

In the current study seven SNPs were analyzed: *IFNG* +874T/A; *IFNG* +2109A/G; *IL12B* +1188A/C; *TNF* −308G/C; *IL17A* rs7747909; *IL10* −819C/T; and *TGFB1* +29C/T. These polymorphisms were selected after verifying their presence in high frequency (5%) in the Brazilian population.

For the current study, 5 mL of peripheral blood was drawn in an EDTA blood tube (by standard phlebotomy procedures), from PTB patients (*n* = 31) and controls (*n* = 20) at base line (T_0_), and genomic DNA was extracted from leukocytes employing a DNAzol commercial reagent (Invitrogen, Carlsbad, CA, USA), according to the manufacturer's instructions. Quantification and purity of the extracted DNA were determined on a spectrophotometer (NanoDrop 2000 Thermo Fisher Scientific). Amplification of the genomic regions of interest was performed by PCR using 20 to 50 ng of DNA, recombinant Taq DNA polymerase, 0.2 mM of each dNTP (deoxy-nucleotide-adenine, guanine, thymine or cytosine-triphosphate), 0.3 to 1 mM concentration of each of the specific primers, appropriate buffer, and ultrapure water. The *IFNG* +874T/A was genotyped by PCR-ARMS as described by Pravica et al. [20]. The variation at *IFNG* +2109A/G creates a restriction site for the enzyme *AciI* and was genotyped by a previously reported PCR-RFLP method [21]. The *IL12B* +1188 genotyping was performed using a PCR-RFLP method in accordance with García-González et al. [22]. Fluorescence-based TaqMan technology (Applied Biosystems, Foster City, CA, USA) was applied to produce genotypes of *IL17A* and *TGFB1* polymorphisms according to the manufacturer's instructions. The *TNF* −308A/G genotype was defined using a PCR-RFLP method in accordance with Wilson et al. [23].

### 2.3. Cytokines Gene Expression by Reverse Transcriptase Real Time PCR (RT-qPCR)

To evaluate IFN-*γ*, IL-12, TNF-*α*, IL-17, IL-10, and TGF-*β* mRNA expressions, 20 mL of peripheral blood was drawn by a standard procedure in heparinized blood tubes, at a single time point from controls (*n* = 20) and at three serial time points for PTB patients (*n* = 31), based on the antituberculosis treatment timeline (T1, T2, and T3), as previously defined. Peripheral blood mononuclear cells (PBMCs) were isolated by a Histopaque gradient separation method [24]. The layer rich in lymphocytes and monocytes was aseptically removed and washed twice with PBS for 15 min at 1500 RPM. The cell suspension was resuspended in 1 mL of PBS and the identification and viability of cells were determined by counting with Turk solution (50 *μ*L aliquots of cell suspension with 50 *μ*L of the dye solution at 5%). Total RNA extraction from PBMC (2 × 10^6^ cells/mL) was prepared using Trizol Reagent (Invitrogen, Carlsbad, CA, USA), according to manufacturer's instructions. The relative purity, concentration, and quality of the isolated RNA were determined by spectrophotometry and the ratio of A260–A280 nm exceeded 1.8 for all preparations (NanoDrop1 1000 Spectrophotometer, Thermo Scientific). To ensure complete removal of traces of genomic DNA, 1 *μ*g of total RNA was incubated with DNase I (Amp Grade).

 First-strand cDNA synthesis was performed with 1 *μ*g of total RNA per 60 *μ*L of reaction using Reverse Transcriptase Super Script II (Invitrogen, Carlsbad, CA, USA) and random primers (3 *μ*g/*μ*L) (Invitrogen, Carlsbad, CA, USA) according to manufacturer's instructions. Within 5 minutes after RNAse H (Invitrogen, Carlsbad, CA, USA) was added and incubated at 37°C for 20 minutes.

Relative quantification of each target mRNA was performed using a standard curve-based method for relative real-time PCR data processing with a 7300 Real-Time PCR Systems (Applied Biosystems, USA) and Power SYBR Green PCR Master Mix [25]. Primers sets used in the qPCR (amplifying cytokine fragments of mRNA and of the human *β*-actin mRNA, endogenous mRNA control) are presented in Table 1. Each q-PCR was set in duplicate in a total of 20 mL each, which contained 0.2 mM of each forward and reverse primer, 2 mL of template cDNA, 10 *μ*L qPCR master mix, and 7.2 mL nuclease-free water. In addition, a “no template” control was included in duplicate on each plate to verify that amplicon contamination was absent. PCR conditions were as follows: initial denaturation at 95°C for 10 min and 40 cycles at 95°C for 15 s and 60°C for 60 s, followed by a melting curve. Amplification of specific transcripts was confirmed by melting curve profiles generated at the end of each run. Control samples expression mean received the relative value of 1.0 and concentrations in all other samples were normalized proportionately.

### 2.4. Plasma Cytokine Levels

Plasma samples were obtained from the same peripheral blood used for the genetic expression of cytokines from controls (*n* = 20) and at three serial time points from patients with PTB, based on the antituberculosis treatment timeline (T1, T2, and T3), as previously defined. Samples were maintained frozen (−80°C) until use and then thawed at room temperature on the day they were used. Quantikine ELISA kits (R & D Systems) were used, according to manufacturer's instructions, to measure IFN-*γ*, IL-12, TNF-*α*, IL-17, IL-10, and TGF-*β* plasma levels and method sensitivity was in accordance with each kit. Cytokine analysis was not possible in all 31 PTB patients because samples were also used in other experiments; therefore the distribution of individuals among cytokines was IFN-*γ* (*n* = 30): T1 (*n* = 5); T2 (*n* = 1); T3 (*n* = 2); with two time points T1 and T2 (*n* = 4); with two time points T1 and T3 (*n* = 1); and with the three time points T1, T2, and T3 (*n* = 17); IL-12, IL-17, and IL-10 (*n* = 30): T1 (*n* = 7); T2 (*n* = 1); T3 (*n* = 2); with two time points T1 and T2 (*n* = 4); and with the three time points T1, T2, and T3 (*n* = 16); TNF-*α* (*n* = 30): T1 (*n* = 5); T2 (*n* = 1); T3 (*n* = 2); with two time points T1 and T2 (*n* = 4); and with the three time points T1, T2, and T3 (*n* = 18); TGF-*β* (*n* = 29): T1 (*n* = 5); T2 (*n* = 1); T3 (*n* = 2); with two time points T1 and T2 (*n* = 4); and with the three time points T1, T2, and T3 (*n* = 17).

### 2.5. Statistical Analysis

Comparisons between different genotypes in the control and patient groups were made using Mann-Whitney *U* Test with two-tail *P* value. For the analytical comparison between the three time points of the treatment in the PTB case group (T1, T2, and T3), a Friedman test was used to verify which time point differed from the other, a Dunn's multiple comparisons test was applied as a posttest. Results were considered significant when *P* < 0.05. Tests were performed using GraphPad Prism version 5.00 for Windows, GraphPad Software (San Diego, CA, USA), http://www.graphpad.com/.

## 3. Results

### 3.1. Demographic Characteristics of PTB Patients

 Gender and age distribution among PTB patients was 23 males (mean age of 48.8 years) and 8 females (mean age of 38.9 years) and all patients were BCG (Bacillus Calmette-Guérin) vaccinated in childhood. Despite the large differences of age and sex in controls and PTB patients groups, these variables had no effect on the cytokines expression and plasma levels (data not shown).

PTB patients' diagnosis was confirmed by sputum smear or culture positivity for *M. tuberculosis* (*n* = 2), sputum smear or culture positive for *M. tuberculosis*, and image exams (radiography or CT) compatible with active TB (*n* = 19) or by image exams (radiography or CT) compatible with active TB (*n* = 10) alone with diagnosis confirmation with clinical response after the beginning of the anti-TB treatment. All patients had respiratory symptoms consistent with dyspnea, cough, and ventilation-dependent pain, and most presented with constitutional symptoms, consistent with weight loss, fever, and weakness at the beginning of the anti-TB treatment (T1). The symptoms were less frequent along treatment timeline (T2) and at the end (T3) all patients were considered recovered. Studies classify pulmonary tuberculosis by its severity as minimal, moderate, or advanced disease, depending on the characteristics of clinical-epidemiologic data and/or image exams (radiography or CT) [16, 19]. In our study medical evaluation of these characteristics showed that all of our patients had a moderate form of active TB disease.

### 3.2. General Immune Response during Anti-TB Treatment

In general, when compared to controls, plasma levels of IFN-*γ*, IL-12, and IL-10 were similar among PTB patients during anti-TB treatment. IL-17 and TGF-*β* plasma levels were increased among PTB patients only at the beginning of anti-TB treatment (T1) and plasma levels for TNF-*α* were lower among PTB patients during anti-TB treatment (T1, T2, and T3) (data not shown). When compared to controls, mRNA expression levels of IL-12, TNF-*α*, and IL-17 were similar among PTB patients during anti-TB treatment (T1, T2, and T3). IFN-*γ* and IL-10 were increased among PTB patients during anti-TB treatment (T1, T2, and T3) and TGF-*β* was increased only at the T2 time point among PTB patients (data not shown).

### 3.3. Influence of *IFNG* +874T/A and +2109A/G Gene Polymorphisms on IFN-*γ* Plasma Level and mRNA Expression

**Figure 1:**
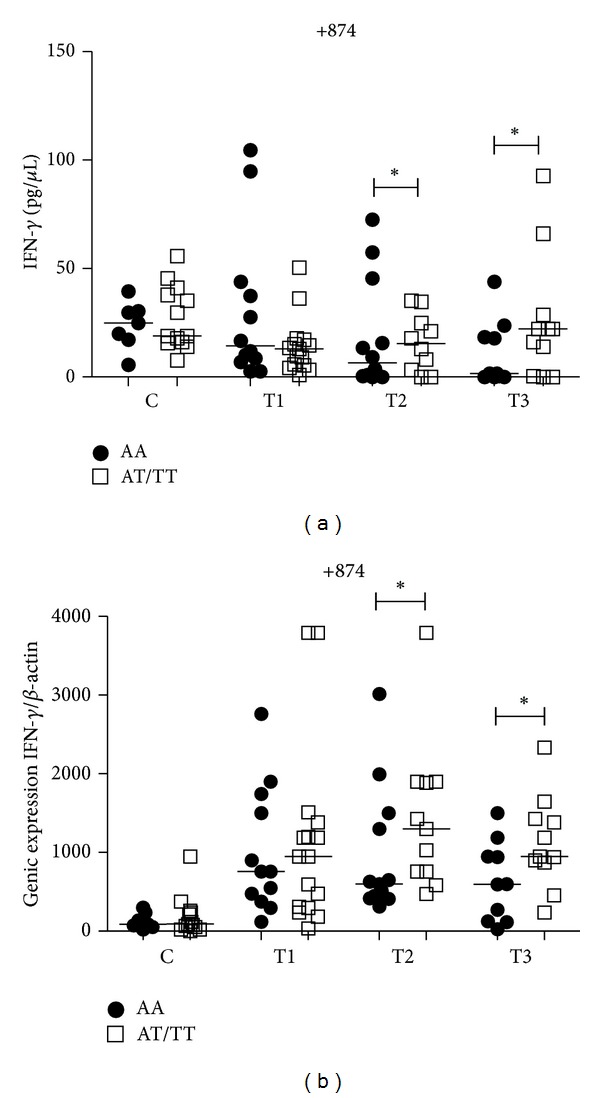
Influence of *IFNG* +874T/A gene SNP on IFN-*γ* plasma levels (a) and mRNA expression (b) in the control group (C) and PTB patients group at three time points of the treatment: T1 (after diagnosis and first month), T2 (three months), and T3 (six months). Data are shown as median levels. *P < 0.05.

The T allele carriers for* IFNG* +874T/A (AT/TT) in PTB patients were associated with significantly higher plasma and mRNA expression levels of IFN-*γ* when compared to individuals with AA genotype at T2 (*P* = 0.04; *P* = 0.03, resp.) and T3 (*P* = 0.04; *P* = 0.03) time points of the treatment (Figures 1(a) and 1(b)). When we compared the three time points, the AA genotype in PTB patients at T1 presented significant higher plasma levels than T2 (*P* < 0.05) and T3 (*P* < 0.05) (Figure 8(a)) and no differences for mRNA expression (Figure 8(b)). T allele carriers at T2 presented with significantly higher mRNA expression levels than at T1 (*P* < 0.05) (Figure 8(b)) and no differences at the IFN-*γ* plasma level (Figure 8(a)) were seen. There was no influence of this polymorphism on the control group (Figures 1(a) and 1(b)).

**Figure 2:**
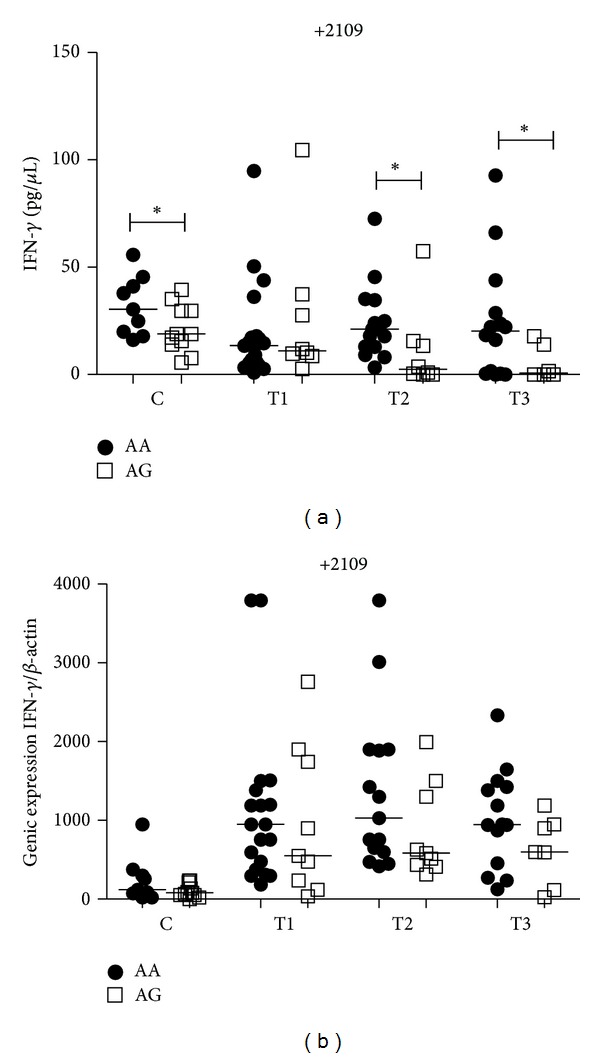
Influence of *IFNG* +2109A/G gene SNP on IFN-*γ* plasma levels (a) and mRNA expression (b) in the control group (C) and PTB patients group at three time points of the treatment: T1 (after diagnosis and first month), T2 (three months), and T3 (six months). Data are shown as median levels. *P < 0.05.

Results for the *IFNG* +2109A/G SNP showed that individuals with AA genotype presented higher levels of IFN-*γ* than the AG genotype in controls (*P* < 0.05) and in PTB patients at T2 (*P* = 0.04) and T3 (*P* = 0.02) (Figure 2(a)). Comparisons between the heterozygous AG genotype of PTB patients time points, using the same individuals, showed that at T1 significantly higher levels of IFN-*γ* were seen than at T2 (*P* < 0.05) and T3 (*P* < 0.05). There were no differences between treatment time points for PTB patients with the AA genotype (Figure 8(c)). No significant differences in mRNA expression were seen between genotypes in the control group and in PTB patients, although patients with the AA genotype tended to present with higher expression levels when compared to individuals with the AG genotype (Figure 2(b)). There were also no differences between treatment time points of PTB patients for mRNA expression within both genotypes (data not shown). In our study group we did not find individuals with the GG genotype.

### 3.4. Influence of *IL12B* +1188A/C Gene Polymorphism on IL-12 Plasma Level and mRNA Expression

**Figure 3:**
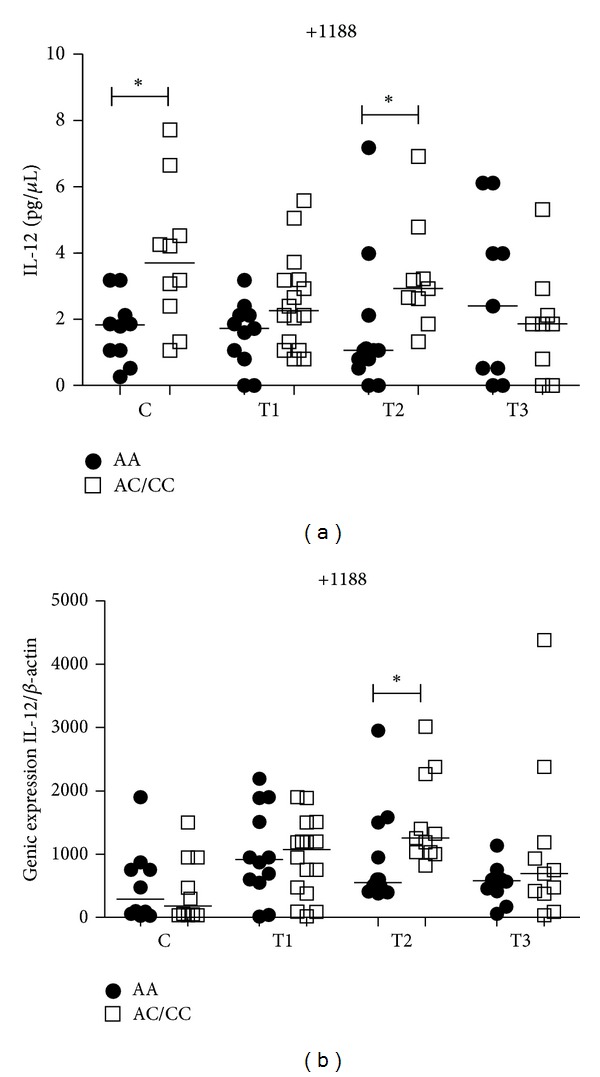
Influence of *IL12B* +1188A/C gene SNP on IL-12 plasma levels (a) and mRNA expression (b) in the control group (C) and PTB patients group at three time points of the treatment: T1 (after diagnosis and first month), T2 (three months), and T3 (six months). Data are shown as median levels. *P < 0.05.

The C allele carriers for *IL12B* +1188 (AC/CC) had significantly higher plasma levels of IL-12 than individuals with the AA genotype in the control group (*P* = 0.04) and at the T2 (*P* = 0.03) time point of PTB patients (Figure 3(a)). IL-12 mRNA expression analysis showed no difference when AA genotype and C allele carriers were compared in the control group. However, at the T2 time point for PTB patients, C allele carriers expressed more IL-12 mRNA expression levels than individuals with AA genotype (*P* < 0.05) (Figure 3(b)). There were also no differences between time points of PTB patients for IL-12 plasma and mRNA expression levels within both genotypes (data not shown).

### 3.5. Influence of *TNF* −308G/C Gene Polymorphism on TNF-*α* Plasma Level and mRNA Expression

**Figure 4:**
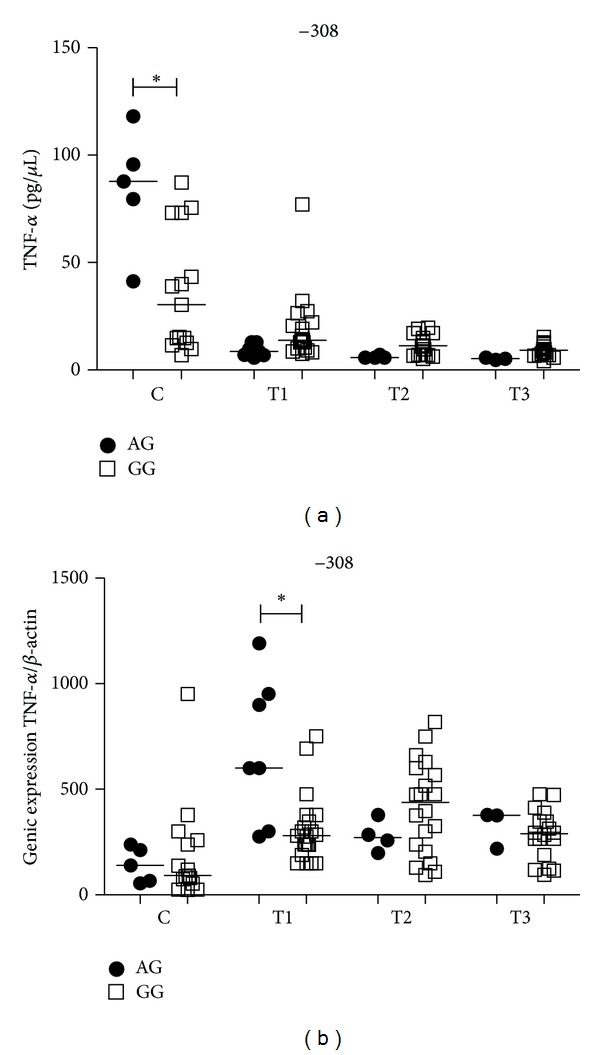
Influence of *TNF* −308G/C gene SNP on TNF-*α* plasma levels (a) and mRNA expression (b) in the control group (C) and PTB patients group at three time points of the treatment: T1 (after diagnosis and first month), T2 (three months), and T3 (six months). Data are shown as median levels. *P < 0.05.

Control individuals with the AG genotype had significantly higher plasma levels of TNF-*α* than those with GG genotype (*P* = 0.04). There was no difference between genotypes in the PTB patients group (Figure 4(a)). The analyses between time points for the GG genotype in PTB patients showed higher levels of TFN-*α* at T1 when compared with T3 (*P* < 0.05). No differences were seen between time points for the AG genotype PTB patients (Figure 8(d)). The *TNF*-308G/C gene SNP did not influence TNF-*α* mRNA expression in controls (Figure 4(b)). At T1, PTB patients with the AG genotype had significantly higher mRNA expression levels than those patients with the GG genotype (*P* = 0.02) (Figure 4(b)). There were also no differences between time points of PTB patients for mRNA expression with either genotype (data not shown). In our study group we did not find individuals carrying the AA genotype.

### 3.6. Influence of *IL17A* rs7747909 Gene Polymorphism on IL-17A Plasma Level and mRNA Expression

**Figure 5:**
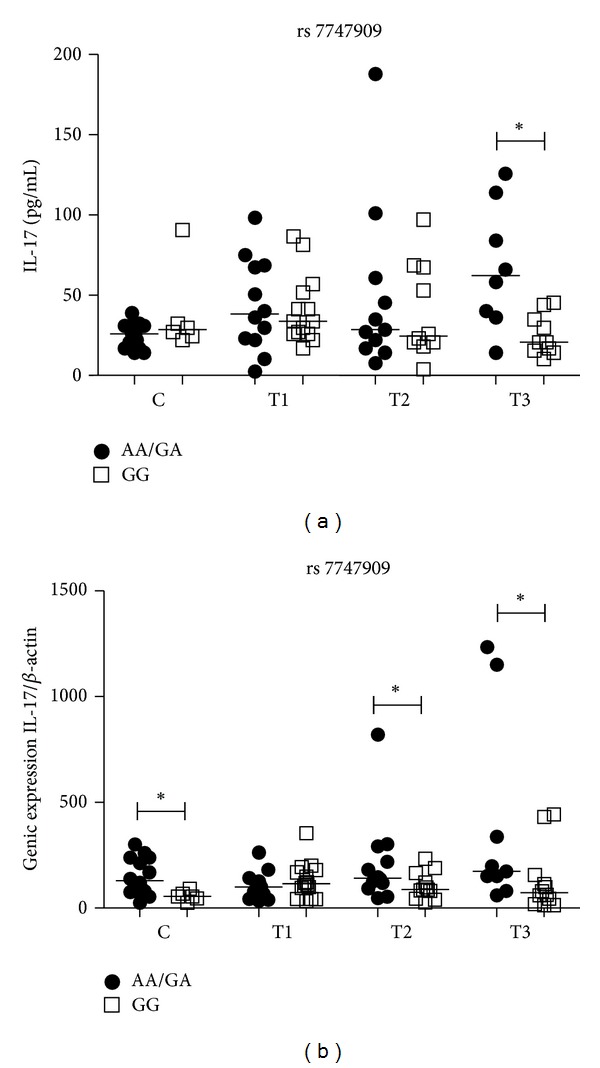
Influence of *IL17* SNP rs7747909 on IL-17 plasma levels (a) and mRNA expression (b) in the control group (C) and PTB patients group at three time points of the treatment: T1 (after diagnosis and first month), T2 (three months), and T3 (six months). Data are shown as median levels. *P < 0.05.

The *marker* rs7747909 at *IL17* gene had no influence on IL-17 control plasma levels. PTB patients who were A allele carriers (AA/AG) had significantly higher IL-17 plasma levels only at the T3 time point (*P* = 0.04) (Figure 5(a)). In controls, the A allele carriers had significantly higher IL-17 mRNA expression levels than individuals with the GG genotype (*P* = 0.04). In PTB patients who were A allele carriers we found significantly higher IL-17 mRNA expression levels at the T2 (*P* < 0.05) and T3 (*P* = 0.04) time points than patients with the GG genotype (Figure 5(b)). There were no differences between time points of PTB patients for IL-17 plasma and mRNA expression levels within both genotype groups (data not shown).

### 3.7. Influence of *IL10* −819C/T Gene Polymorphism on IL-10 PLasma Level and mRNA Expression

**Figure 6:**
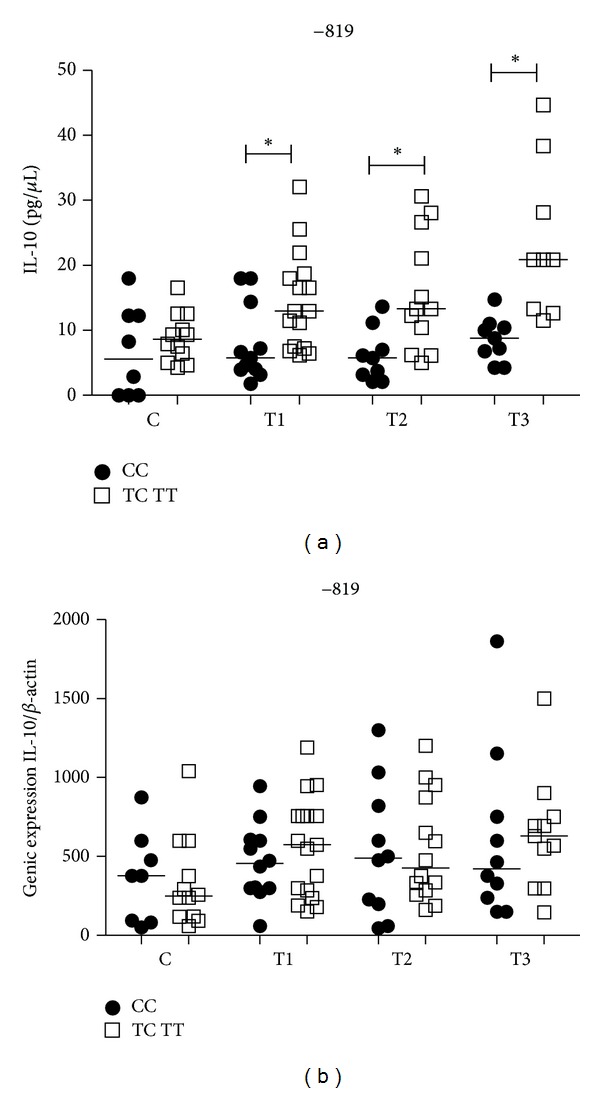
Influence of *IL10* −819C/T gene SNP on IL-10 plasma levels (a) and mRNA expression (b) in the control group (C) and PTB patients group at three time points of the treatment: T1 (after diagnosis and first month), T2 (three months), and T3 (six months). Data are shown as median levels *P < 0.05.

 There was no difference in IL-10 plasma levels in the controls between CC genotype and T allele carriers. PTB patients who were carriers of the T allele (TC/TT) had significantly higher levels of IL-10 when compared with PTB patients with the CC genotype in all time points of treatment T1 (*P* < 0.05), T2 (*P* = 0.03), and T3 (*P* < 0.05) (Figure 6(a)). IL-10 −819C/T SNP had no influence on IL-10 mRNA expression in the control group and PTB patients when the CC genotype and T allele carriers were compared (Figure 6(b)). There were also no differences between time points in PTB patients for IL-10 plasma and mRNA expression levels within both genotypes (data not shown).

### 3.8. Influence of *TGFB1* +29C/T Gene Polymorphism on TGF-*β* Plasma Level and mRNA Expression

**Figure 7:**
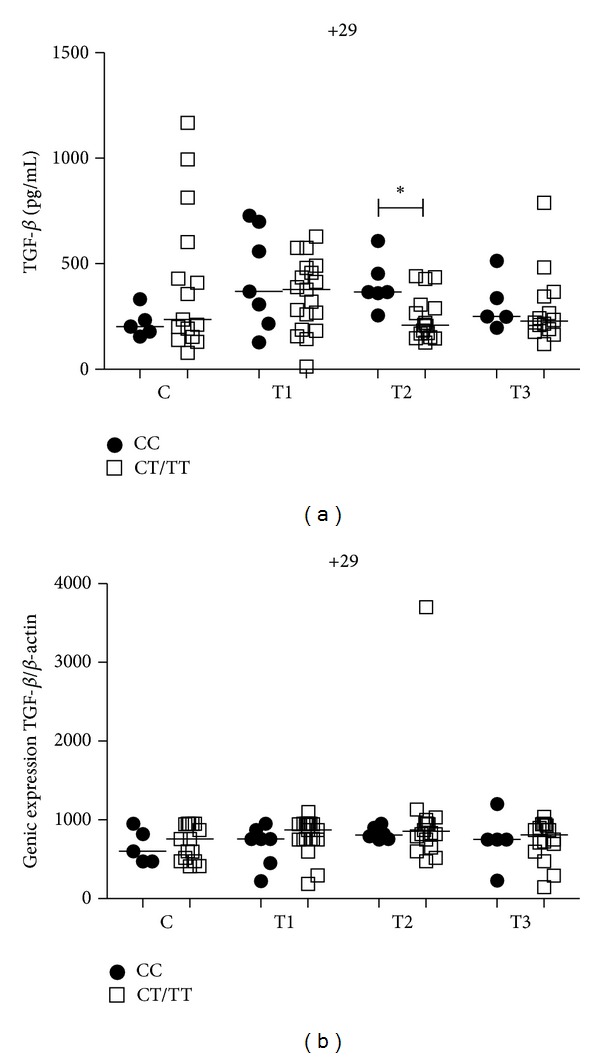
Influence of *TGF*β*1* +29C/T gene SNP on TGF-*β* plasma levels (a) and mRNA expression (b) in the control group (C) and PTB patients at three time points of the treatment: T1 (after diagnosis and first month), T2 (three months), and T3 (six months). Data are shown as median levels. *P < 0.05.

**Figure 8:**
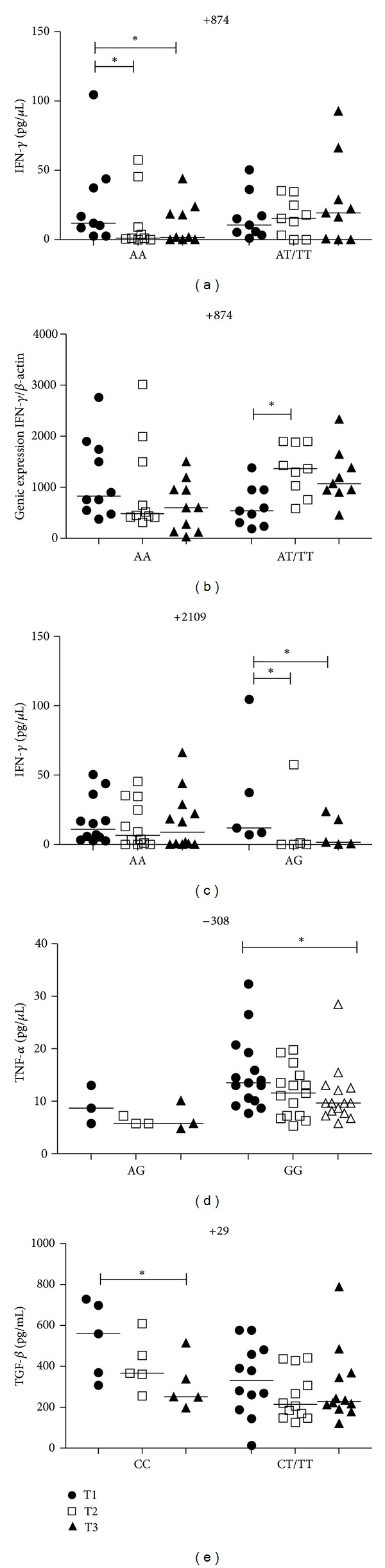
Influence of *IFNG* +874T/A gene SNP on IFN-*γ* plasma levels (a) and mRNA expression (b), *IFNG* +2109A/G gene SNP on IFN-*γ* plasma levels (c), *TNF* −308G/C gene SNP on TNF-*α* plasma levels (d), and TGFB1 +29C/T gene SNP on TGF-*β* plasma levels (e) in PTB patients that have all three time points of the treatment: T1 (after diagnosis and first month), T2 (three months), and T3 (six months). Data are shown as median levels. *P < 0.05.

No differences in TGF-*β* plasma levels were seen in the control group between both genotypes. PTB patients with CC genotype had higher TGF-*β* plasma levels when compared with T allele carriers only at the T2 time point (*P* = 0.02) (Figure 7(a)). Analyses between time points of PTB patients with CC genotype showed higher TGF-*β* plasma levels at T1 when compared to T3 (*P* < 0.05) (Figure 8(e)). No differences in mRNA expression were seen in both controls and PTB patients within both genotypes (Figure 7(b)). There were also no differences between time points of PTB patients for mRNA expression within both genotypes (data not shown).

## 4. Discussion

It is well known that cytokines play an essential role during the immune response to active TB disease and changes in cytokine levels may lead to abnormal or ineffective immune responses, as seen in human infections caused by *M. tuberculosis* [6]. The genetic component that contributes to susceptibility and progression of PTB most certainly involves an interaction between multiple alleles located on different genes [26]. As a number of cytokine genes have been described in association with altered cytokine levels [13, 20], we evaluated the influence of SNPs at cytokine genes on the overall cytokine response of PTB patients undergoing anti-TB treatment. When pertinent, controls results were considered to indicate an effect of the polymorphism under normal conditions, without the interference of active TB or anti-TB treatment, in other words, an independent genetic effect. 

Our results showed that PTB patients who were T allele carriers had higher plasma and mRNA expression levels of IFN-*γ* at the middle and end of anti-TB treatment. Studies have shown that the *IFNG* +874A/T SNP influences IFN-*γ* production by providing a binding site for NF-*κ*B and is associated with TB susceptibility [14, 15, 20]. This fact could have functional consequences for the transcription of IFN-*γ* production [27, 28]. 

A recent study in Spain showed that the *IFNG* +874 AA genotype had the lowest IFN-*γ* production in PBMC culture after stimulation with PPD in PTB patients at the time of diagnosis and after completion of therapy [14], which agrees with our results at the end of anti-TB treatment. Still in agreement with our results, 2 additional studies have shown that the homozygous TT genotype of normal and PTB individuals produces higher IFN-*γ* in response to mycobacterial antigens [15, 16]. In another functional study patients with tuberculosis carrying the genotype +874AA showed significantly lower IFN-*γ* plasma levels than those with the +874AT and +874TT genotypes [11]. This trend was also seen in the plasma of Indians patients with active PTB [12].

Other studies that evaluated IFN-*γ* serum levels in HBV infection and IFN-*γ* production in PBMC cultures in acutely ill patients, cutaneous leishmaniasis, and normal individuals stimulated with LPS (Lipopolysaccharide), PHA (Phytohemagglutinin), or *Mycobacterium leprae* (*M. leprae*) antigens also support our findings of higher IFN-*γ* levels in PTB patients that were T allele carriers (TA/TT) [29–31]. Another study has shown the relation between an *IFNG* polymorphic microsatellite marker (allele 2) polymorphism and the IFN-*γ* gene transcription. Since this region shows an absolute correlation with the *IFNG* +874 T allele, this study's findings are in agreement with our results, that showed that the *IFNG* +874 T allele was related to a higher IFN-*γ* expression from the middle to the end of the anti-TB treatment [20]. 

Some studies evaluating tuberculosis patients in the initial stages of TB disease using normal controls have found no differences between genotypes of *IFNG* +874 SNP in IFN-*γ* production, in PBMC cultures of *M. tuberculosis* H37Rv, culture filtrate antigen (CFA) of *M. tuberculosis* nor PHA stimulus [17, 18]. An additional study found no influence of *IFNG* +874 locus on mRNA expression during *Helicobacter pylori* (*H. pylori*) infection [32]. These different findings may be due to ethnic differences in genotypic frequencies among various population studies. This result however is in complete agreement with genetic epidemiologic data that the T allele is associated with protection against TB [33]. This same allele is also associated with resistance to leprosy [34].

Another polymorphism in the *INFG* gene located 2,109 bp downstream from the translation start site in the third intron has been reported to be involved in transcriptional regulation of IFN-*γ* gene of this polymorphism, although the contribution is still unclear [35, 36]. To our knowledge our study is the first report regarding the functional effect of *INFG* +2109A/G on IFN-*γ* plasma and mRNA expression levels, as our results showed that controls and PTB patients with the AG genotype had lower plasma levels at the end of the treatment.

IFN-*γ* is a key cytokine in activation of macrophages for mycobacterial stasis and killing [2]; the persistence of low IFN-*γ* production from the middle to end of therapy in patients with *IFNG* +874 AA genotype and *INFG* +2109 AG genotype could result in a worse prognosis to the resolution of the active disease and efficiency of the anti-TB treatment and may also underlie their increased risk for reactivation of a latent PTB focus. In agreement with our results, decreased production of IFN-*γ* in PTB patients when compared with healthy controls have been reported and it may be due to the initial T cell anergy seen in the disease [18, 37–39]. Such an inadequate IFN-*γ* production may result in failure of macrophage activation, which could lead to active disease progression and could play a role affecting TB diagnostic tests, as *IFG* alleles were associated with microscopy-positive/negative and bacterial culture-positive/negative forms of disease [18, 40].

IL-12 is important in mediating protective immunity against TB. An SNP at 3'UTR region of the *IL12B* gene (+1188A/C) coding for IL-12p40 is known to modulate IL-12p40 levels. The present study showed that *IL12B* +1188 AA genotype is associated with lower IL-12 plasma levels in normal controls and in TB patients after 3 months of anti-TB treatment. Our results agree with another study that evaluated PTB patients and normal controls [17].

Several studies have found that the *IL12B* +1188 C allele is associated with lower IL-12p40 production of PBMC from healthy individuals stimulated with C3 binding glycoprotein, LPS, or PPD [41–43]. Arababadi et al. [30] did not find a significant difference in IL-12 serum level between AA and AC genotypes in occult HBV infection. Discordance between results may be due to differences in ethnicity, diseases, and designs of the studies.

IL-12 production is induced following phagocytosis of *M. tuberculosis* by macrophages and dendritic cells, which leads to development of a Th1 response with production of IFN-*γ* [1]. Since IL-12p40 is a component of both IL-12p70 and IL-23 and regulates initiation and maintenance of acquired cellular responses to TB, low IL-12p40 levels in AA genotype individuals might have a role in limiting chronic inflammation [44], which could result in more difficulties in resolving active disease and dampen the efficiency of the anti-TB treatment.

TNF-*α* plays an important role in granuloma formation in tuberculosis [19]. The promoter region of the *TNF* gene is highly polymorphic and our evaluation of the *TNF* −308G/A *locus* showed that normal individuals with the AG genotype had higher TNF-*α* plasma levels. PTB patients carrying the same genotype tended to have higher levels of TNF- *α*, though not significant. There are conflicting results regarding the functional effect of the *TNF* −308A/G SNP. Some authors have demonstrated increased TNF-*α* production related to *TNF* −308 A allele carriers in LPS stimulated PBMC and whole blood cultures of leprosy patients after LPS and *M. leprae* stimulation and of paracoccidioidomycosis patients, while others failed to show any effect of this SNP on TNF-*α* production after LPS stimulation *in vivo* or *in vitro*, in HCV patients and in tuberculosis patients [19, 44–50].

An *in vitro* expression study has indicated that the *TNF −308G/A* SNP has a direct effect on TNF-*α* gene regulation, and the A allele at this locus may lead to a higher expression level [51]. Our results agree with this study that demonstrated that PTB patients with the AG genotype at the beginning of anti-TB treatment have higher TNF-*α* mRNA expression than GG genotype individuals.

Since TNF-*α* is important for walling off infections and preventing dissemination by granuloma formation, low levels of TNF-*α*, as seen in our PTB patients, could impair the containment of the *M. tuberculosis* bacilli, leading to difficult resolution of active disease, dampening the efficiency of anti-TB treatment and increasing the reactivation risk, as shown in rheumatoid arthritis patients who were undergoing anti-TNF-*α* therapy [52, 53].

IL-17 is a potent inflammatory cytokine induced by *M. tuberculosis *infection [54]. To our knowledge this is the first study to verify the functional effect of the *IL17A* rs7747909 polymorphism on IL-17A plasma and mRNA expression levels. Our results showed that A allele carriers (at rs7747909) produce higher plasma levels of IL-17A at the end of therapy and, in general, PTB patients produced higher levels than normal controls. 

Although Th17 cells are not as important as Th1 cells in mediating protection against primary *M. tuberculosis* infection, IL-17 appears to be critical to the induction of *M. tuberculosis*-specific memory response and the mediation of protection against challenge infections and during vaccinations [55–57]. Our results suggest that PTB patients have no impairment to IL-17A production.

IL-10 is known to have deactivating properties and undermines human Th1 immunologic responses. About 50% of the observed variability of IL-10 secretion can be explained by genetic factors [58]. Our results showed that *IL10* −819 T allele carriers had higher plasma levels of IL-10 during the 6 months of anti-TB treatment. A previous study evaluating PTB patients showed no influence of this SNP on IL-10 levels [17]. Additionally, no impact of the *IL10* −819 locus was found on IL-10 serum level of controls and patients with HCV infection or on normal individuals PBMC cultures stimulated with LPS and PPD [13, 48]. A study on patients with leprosy showed that *IL10* −819 T allele carriers produce lower levels of IL-10 when compared with non-T allele carriers [59].

In a recent study evaluating *H. pylori*, patients with SNPs in the promoter region of *IL10* were shown to have higher IL-10 mRNA expression with the GCC haplotype carriers at *IL10* −*1082G*/*A*/−*819CT*//−*592C*/*A* and low IL-10 mRNA expression levels in ATA haplotype carriers [32]. In our study the different *IL10* −*819* genotypes had no influence on IL-10 mRNA expression.

Published data suggests that IL-10 inhibits synthesis of IFN-*γ* by T cells and that production of IL-10 has been associated with anergy in tuberculosis [60, 61]. Our results suggest that PTB patients T allele carriers could have more difficulties in building a protective response towards active PTB since they are present with higher levels of IL-10 during anti-TB treatment. These results suggest that in cases with advanced forms of PTB, higher IL-10 production in T allele carries could lead to a worse recovery from active disease and poor anti-TB treatment efficiency.

TGF-*β* is present in the granulomatous lesions of TB patients [62]. At low concentrations TGF-*β* is a chemotactic factor for monocytes and acts to induce secretion of IL-1*α* and TNF-*α* and, in high concentration, inactivates macrophages, inhibits the expression and function of receptors for IFN-*γ*, IL-1*α*, and IL-2, and decreases the production of TNF-*α*, parallel events related to increase of intracellular mycobacterial growth [63, 64]. Our results showed that PTB patients with the *TGFB1* +21CC genotype have higher TGF-*β* plasma levels at T2 of the anti-TB treatment. These results are consistent with other studies focused on cancer and myocardial infarction [65–67]. Our results revealed that TGF-*β* plasma levels were higher at the beginning of the anti-TB treatment in all PTB patients when compared to the controls, which could result in the impairment of the initial protective immune response for the resolution of the active disease and in depressing the efficiency of anti-TB treatment. The fact that PTB patients with the *TGFB1* +21 CC genotype also maintained higher levels of the cytokine at T2 could result in a more persistent active disease.

Our work lacks an association between demographic characteristics and cytokine levels. In addition, the influence of cytokine SNPs on TB outcomes may be driven by a small sample size and by the fact that all patients had a moderate presentation of PTB. We also had differences between the plasma levels patterns and mRNA expression levels patterns of the cytokines evaluated in the controls and TB patients during anti-TB treatment. This fact could be explained by mRNA stability and transcription rate and by factors of translational regulation which could directly affect the expression and production of mediators involved in immune response [68].

The present study suggests that *IFNG* +874T/A, *IFNG* +2109A/G, *IL12* +1188A/C, *IL10* −819C/T, and *TGFB1* +21C/T are associated with different cytokine levels in PTB patients and may play a role in the initiation and maintenance of acquired cellular immunity to TB and in the outcome of the active disease and antituberculosis treatment. As cytokines play a major role in TB immunity, studying the overall cytokine profile determined by the respective functional SNPs and/or other closely linked genes could demonstrate the actual pattern of the cytokine response against the mycobacteria and may provide a better understanding of active TB disease progression and response to the anti-TB treatment and may serve as genetic risk markers of TB susceptibility.

## 5. Conclusion

In this study we demonstrated that cytokine SNPs can induce different overall cytokine levels in PTB patients during anti-TB treatment and these levels could be important to the outcome of the treatment. Future studies with a larger population and different forms and severity stages of tuberculosis will help to better understand why many individuals are infected by the mycobacterial bacilli but only 10% develop active disease.

## Figures and Tables

**Table 1:** Primers sequence for qPCR.

Primers	Forward sequence	Reverse sequence
IFN-*γ*	5′-AAAAGAGTTCCATTATCCGCTACATC-3′	5′-GTTTTGGGTTCTCTCTTGGCTGTTA-3′
IL-12	5′-ACCTCCACCTGCCGAGAAT-3′	5′-CATGGTGGATGCCGTTCA-3′
TNF-*α*	5′-GGTTTGCTACAACATGGGCTACA-3′	5′-CCCCAGGGACCTCTCTCTAATC-3′
IL-17	5′-TTAGGC ACATGGTGGACAATCGG-3′	5′-ATGACTCCTGGGAAGACCTCA TTG-3′
IL-10	5′-CTTGATGTCTGGGTCTTGGTTCT-3′	5′-GCTGGAGGACTTTAAGGGTTAACCT-3′
TGF-*β*	5′-AGGGCCAGGACCTTGCTG-3′	5′-CAAGGGCTACCATGCCAACT-3′
*β*-actin	5′-GCTGGAAGGTGGACAGCGA-3′	5′-GGCATCGTGATGGACTCCG-3′
